# Impact of Residents’ Mass Resignation in Cardiovascular Surgery: A System Sustainability Perspective

**DOI:** 10.1093/icvts/ivag037

**Published:** 2026-02-06

**Authors:** June Yeop Lee, Hyoung Woo Chang, Sang Yoon Kim, Joon Chul Jung, Jae Hang Lee, Sanghon Park, Jun Sung Kim, Kay-Hyun Park

**Affiliations:** Department of Thoracic and Cardiovascular Surgery, Seoul National University Bundang Hospital, Seongnam-si, Gyeonggi-do 13620, Republic of Korea; Department of Thoracic and Cardiovascular Surgery, Seoul National University College of Medicine, Seoul 03080, Republic of Korea; Department of Thoracic and Cardiovascular Surgery, Seoul National University Bundang Hospital, Seongnam-si, Gyeonggi-do 13620, Republic of Korea; Department of Thoracic and Cardiovascular Surgery, Seoul National University College of Medicine, Seoul 03080, Republic of Korea; Department of Thoracic and Cardiovascular Surgery, Seoul National University Bundang Hospital, Seongnam-si, Gyeonggi-do 13620, Republic of Korea; Department of Thoracic and Cardiovascular Surgery, Seoul National University College of Medicine, Seoul 03080, Republic of Korea; Department of Thoracic and Cardiovascular Surgery, Seoul National University Bundang Hospital, Seongnam-si, Gyeonggi-do 13620, Republic of Korea; Department of Thoracic and Cardiovascular Surgery, Seoul National University College of Medicine, Seoul 03080, Republic of Korea; Department of Thoracic and Cardiovascular Surgery, Seoul National University Bundang Hospital, Seongnam-si, Gyeonggi-do 13620, Republic of Korea; Department of Thoracic and Cardiovascular Surgery, Seoul National University College of Medicine, Seoul 03080, Republic of Korea; Department of Thoracic and Cardiovascular Surgery, Seoul National University Bundang Hospital, Seongnam-si, Gyeonggi-do 13620, Republic of Korea; Department of Thoracic and Cardiovascular Surgery, Seoul National University College of Medicine, Seoul 03080, Republic of Korea; Department of Thoracic and Cardiovascular Surgery, Seoul National University Bundang Hospital, Seongnam-si, Gyeonggi-do 13620, Republic of Korea; Department of Thoracic and Cardiovascular Surgery, Seoul National University College of Medicine, Seoul 03080, Republic of Korea; Department of Thoracic and Cardiovascular Surgery, Seoul National University Bundang Hospital, Seongnam-si, Gyeonggi-do 13620, Republic of Korea; Department of Thoracic and Cardiovascular Surgery, Seoul National University College of Medicine, Seoul 03080, Republic of Korea

**Keywords:** education, trainee, doctor strike, resident resignation, failure-to-rescue

## Abstract

**Objectives:**

In February 2024, a nationwide resident resignation occurred in South Korea that persisted for more than one and a half years and caused unprecedented disruptions in teaching hospitals. This study evaluated the clinical and socioeconomic impact of resident absence on cardiovascular surgery at a tertiary teaching hospital.

**Methods:**

We retrospectively reviewed 681 patients who underwent open-heart or aortic surgery between February 20 and November 30, 2023 (before resident absence) and in 2024 (resident absence). Each year was divided into 3 periods (Q1, Q2, and Q3) for temporal comparison. The primary outcomes were 30-day mortality, failure-to-rescue complications, and failure-to-rescue. Failure-to-rescue was defined as in-hospital mortality after one or more of the following failure-to-rescue complications: acute renal failure, respiratory complications (prolonged ventilation >24 h, pneumonia, or tracheostomy), stroke, reoperation, life-threatening arrhythmia, postoperative myocardial infarction, or culture-positive sepsis. Multivariable logistic regression was performed to identify independent risk factors.

**Results:**

When comparing 2023 Q1 with 2024 Q1, surgical volume decreased from 154 to 65 cases (−58%) and did not return to 2023 Q1 baseline. Compared with the 2023 group, the median surgical waiting time of the 2024 group increased from 17 [IQR: 8-28] to 36 [IQR: 20-58] days (*P* < .001). Resident absence was not a risk factor for 30-day mortality but was an independent risk factor for both failure-to-rescue complications (OR 1.50, 95% CI 1.03-2.19, *P* = .035) and failure-to-rescue (OR 3.64, 95% CI 1.33-9.98, *P* = .012).

**Conclusions:**

The nationwide resignation of residents revealed the structural vulnerability of South Korea’s healthcare system, which relies heavily on residents’ workforce. Surgical capacity decreased, waiting times increased, and rescue outcomes deteriorated. The resident-dependent healthcare system requires reform, with teaching hospitals treating residents primarily as trainees rather than as inexpensive labour.

## Graphical abstract

**Figure:**
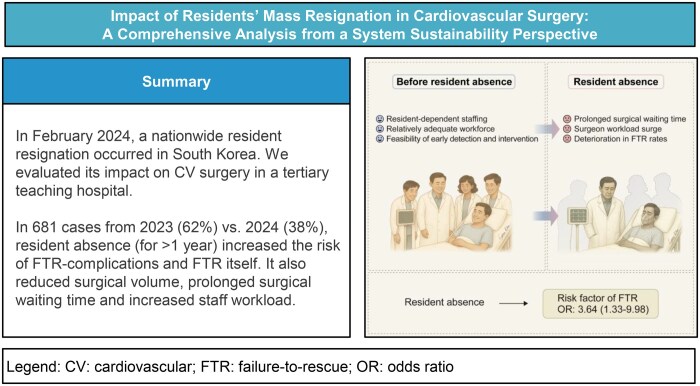

## INTRODUCTION

Residents hold a unique position, serving as both salaried employees and trainees.

In South Korea, 97% of the population is supported by the National Health Insurance Service, and the remaining 3% by medical aid. With low reimbursement rates, individuals pay approximately 3.5% of their income as insurance premiums while receiving high-quality, easily accessible healthcare. The avoidable mortality rate is 142 per 100 000 population (OECD 239; Germany 195; United States 336). The average number of annual outpatient visits per person is 15.7 (OECD 5.9; Germany 9.6; United States 3.4).^1^ Most patients can see a specialist on the same day.

Behind these favourable statistics, however, lies a structural problem. To offset deficits from low reimbursement, teaching hospitals have reduced the number of board-certified specialists and relied heavily on residents. Residents work more than 80 h weekly for near-minimum wages and constitute more than 40% of the physicians in teaching hospitals.^2^^,^^3^ Moreover, even residents in South Korea may face criminal prosecution and actual penalties for adverse clinical outcomes, which has intensified the avoidance of high-risk specialties.^4^^,^^5^

Policymakers have linked this avoidance to a physician shortage, citing a density of 2.7 per 1000 population, below the OECD average of 3.7, and in February 2024, they announced a 65% increase in the medical school admission quota (3058-5058) and regulated non-reimbursable services to attract medical students to high-risk specialties.

Medical professionals warned that sudden expansion without sufficient faculty and infrastructure would compromise education quality and endanger public health. Nevertheless, government and university administrators proceeded. In protest, more than 95% of the residents resigned on February 20, 2024, and most medical students declared indefinite leave; both actions continued through August 2025. Without residents, teaching hospitals cannot sustain existing workloads, resulting in clinical and structural changes.

Resident strikes are rare but have increased since COVID-19, becoming a major social issue. Most studies have reported no short-term increase in mortality, but prolonged nationwide absences are exceptional and unstudied in CV surgery, where challenging surgeries and intensive care are frequently performed.^6^

In this study, the clinical, structural, and socioeconomic impacts of the nationwide mass resignation of residents on CV surgery in a tertiary teaching hospital were evaluated. In addition, the sustainability of a resident-independent system in South Korea was assessed.

## PATIENTS AND METHODS

This study involved the collection and analysis of clinical data only and did not include the storage or use of any biological materials. The handling of collected data was conducted in a manner consistent with the requirements outlined in the WMA Declaration of Taipei. The study protocol was reviewed and approved by the Institutional Review Board of Seoul National University Bundang Hospital (SNUBH; B-2412-941-101). Given the retrospective nature of the study and the use of de-identified data, the requirement for informed consent was waived by the ethics committee.

### Study design and patient selection

We reviewed patients who underwent open-heart surgery or open aortic surgery (collectively OHS) in the CV surgery department at SNUBH from February 20 to November 30 in both 2023 and 2024. February 20, the date of the nationwide residents’ resignation, was chosen as the starting point. Identical periods were selected to minimize seasonal variations.

Data were extracted from a prospectively collected cardiac surgery database. Each year was divided into 3 intervals, that is February-May (Q1), June-August (Q2), and September-November (Q3), to assess temporal changes.

Coronary artery surgery included coronary artery bypass grafting with or without cardiopulmonary bypass. Valve surgery included replacement or repair of the aortic, mitral, or tricuspid valves. Aortic surgery included replacement of any segment from the root to the abdominal aorta, excluding peripheral arteries. Complex surgery involved 2 or more of these procedures during the same operation.

The exclusion criteria were as follows: (1) hospital stay >60 days, (2) percutaneous interventions, and (3) planned staged surgery at the same admission. These were predetermined through discussion among the coauthors, as such cases often have heterogeneous clinical courses.

Revenue data were obtained from the SNUBH information department and reported only as percentage changes due to institutional privacy policy.

### Outcome variables

The primary outcomes were 30-day mortality, failure-to-rescue (FTR) complications and FTR. FTR was defined as in-hospital mortality after ≥1 of the following complications: acute renal failure (excluding preoperative dialysis); respiratory complications (prolonged ventilation >24 h, pneumonia, or tracheostomy); stroke; reoperation (including bleeding control); life-threatening arrhythmia; postoperative myocardial infarction; or culture-positive sepsis. Definitions followed the Society of Thoracic Surgeons Adult Cardiac Surgery Database (STS-ACSD; **Supplementary Material 1**).

Secondary outcomes were readmission, duration of mechanical ventilation, inotropic/vasopressor use, chest tube drainage, intensive care unit (ICU) and hospital stay, and surgical waiting time


$$\mathrm{FTR}=\frac{\#\;\mathrm{of}\;\mathrm{deaths}\;\mathrm{from}\;\mathrm{FTR}\;\mathrm{complications}\;}{\#\;\mathrm{of}\;\mathrm{patients}\;\mathrm{who}\;\mathrm{have}\;\mathrm{FTR}\;\mathrm{complication}(s)\;}$$

### Institutional changes following residents’ resignation

This section summarizes only measures directly related to clinical practice. The timeline and government’s actions are summarized in **Supplementary Materials 2 and 3**.

In 2023, at SNUBH, there were a total of 9 residents in the Department of Thoracic and Cardiovascular Surgery. Of these, 5 residents were assigned to CV surgery and 4 to general thoracic surgery, rotating every 2 months. They participate in surgical assistance, postoperative care, medical record documentation, emergency department coverage, and academic research. All the residents resigned on February 20, 2024, and the chief resident provided a 1-week handover to consultants to ensure continuity of patient care. On March 1, 2024, the contracts of the clinical fellows expired.

The faculty included 1 coronary specialist, 1 valve specialist, 2 aortic specialists, 2 intensivists, and 1 junior consultant; however, their surgical practice was not limited to their specialties. In August 2024, the valve specialist resigned, and the remaining 4 consultants shared valve surgeries. All surgeries, including emergency sternotomy and bleeding control surgery, were performed by faculty surgeons rather than residents in both 2023 and 2024.

In 2023, 5 physician assistants (PAs) and 4 surgical assistants (SAs) worked in CV surgery. After governmental approval to expand nurses’ roles, 3 PAs (August 2024) and 3 SAs (October 2024) were added.

The on-call system also changed. Before resident absence, 2 residents covered the ward and ICU separately, with a fellow or junior consultant on home call as a backup. After the residents’ resignation, one consultant covered both the ward and ICU. Standardized protocols for nonemergent conditions were provided to nurses, and PAs handled primary calls to reduce consultant workload. Three consultants rotated emergency aortic calls both before and after the residents’ absence.

### Statistical analysis

Categorical variables were compared with the chi-square test or Fisher’s exact test, and continuous variables were compared with Student’s *t* test or the Mann-Whitney *U* test. Variables with a *P-*value ≤.1 in the univariable analysis were entered into the multivariable logistic regression. The backwards selection method was used, but variables of interest excluded during the process were nonetheless included in the final model. Multicollinearity was checked via the variance inflation factor (VIF), and variables with a VIF > 5 were excluded. Analyses were performed with SPSS 27.0 for Windows (IBM, Armonk, NY, United States).

## RESULTS

### Preoperative and operative characteristics

**Figure 1.**
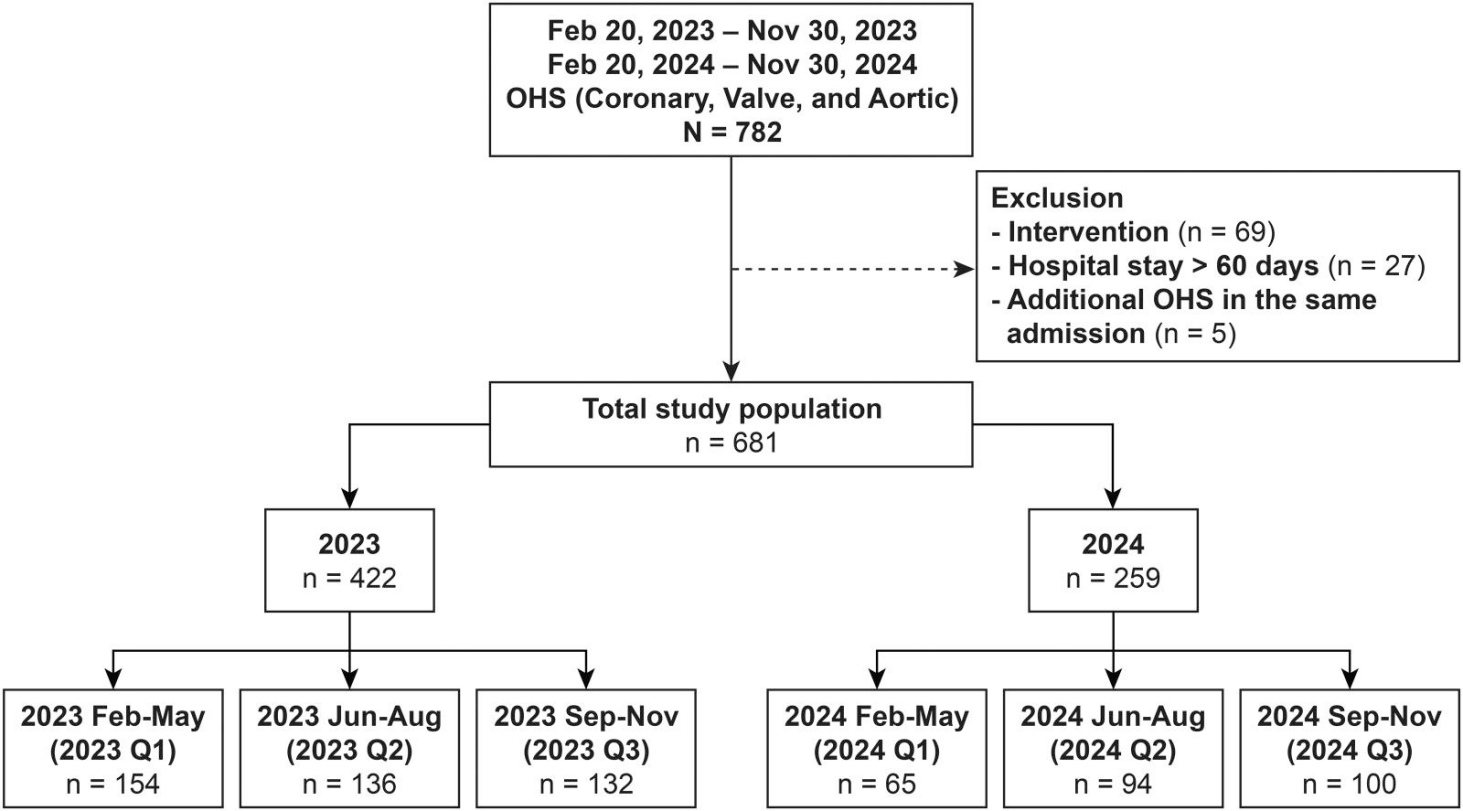
Patient Classification Flowchart of the Study Cohort. Abbreviation: OHS: open-heart surgery.

A total of 681 patients who underwent OHS between 20 February and 30 November in each of 2023 and 2024 were included and analysed according to the year of surgery: 2023 group (*n* = 422) and 2024 group (*n* = 259) (**Figure 1**). Each group was further subdivided into Q1, Q2, and Q3, and comparisons were drawn between corresponding quarters.

**Figure 2.**
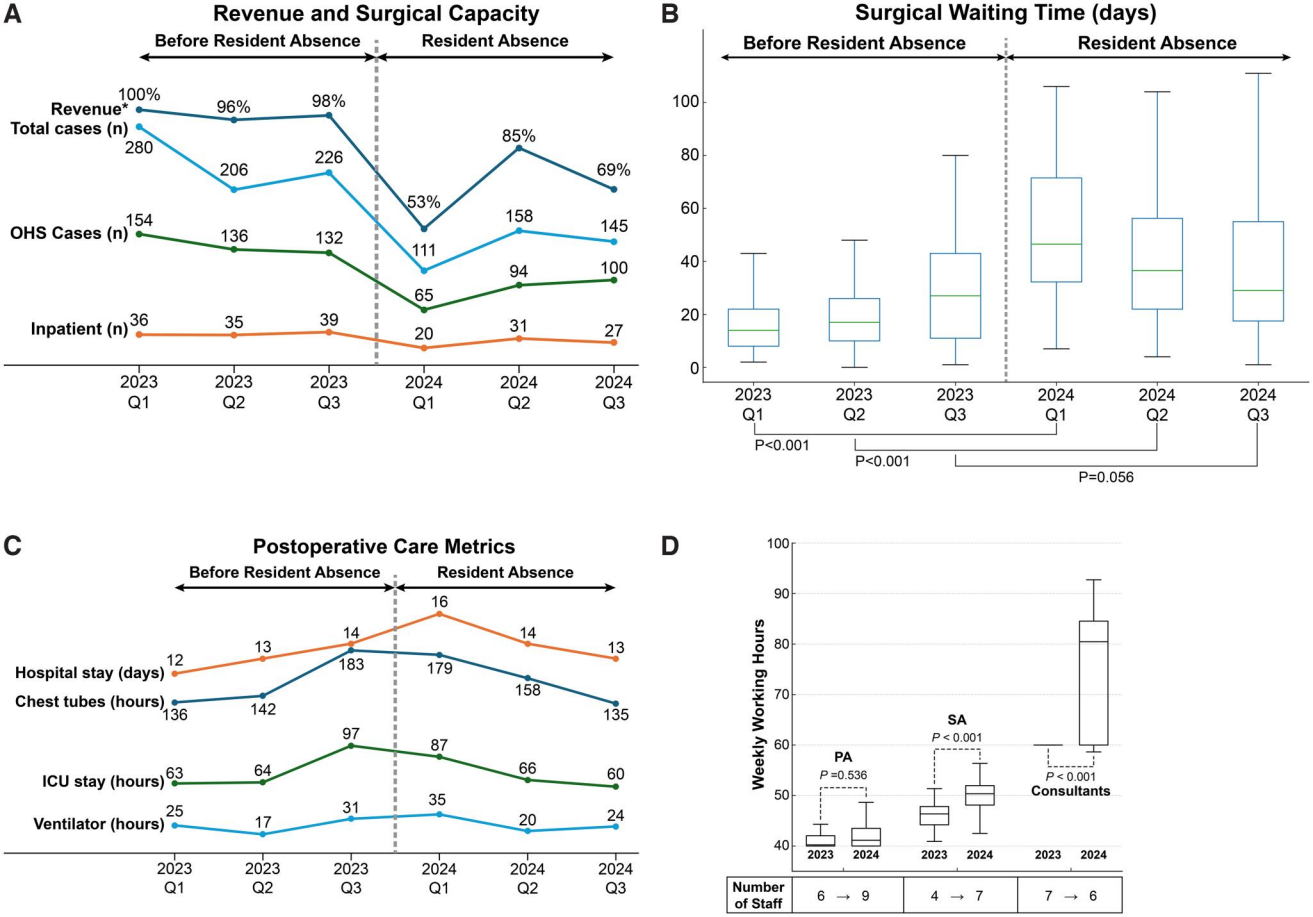
Changes Before and After the Resident Resignation. (A) Line graphs showing changes in revenue and surgical capacity, (B) line graphs showing changes in postoperative care metrics, (C) box plots showing changes in surgical waiting time, and (D) box plots showing changes in weekly working hours of medical staff. Abbreviations: ICU: intensive care unit; OHS: open-heart surgery; PA: physician assistant; SA: surgical assistant. *2023 Q1 revenue = 100% (reference).

The preoperative and operative characteristics are summarized in **Table 1**, with the 2024 group (*n* = 259) representing a 38.6% reduction in case volume compared with 2023 group (*n* = 422) (**Figure 2A**). Most demographics and clinical parameters were comparable, but several differences were noted.

**Table 1.** Comparison of Overall Patient Characteristics Between 2023 and 2024 Groups

	2023 Q1 (*n* = 154)	2024 Q1 (*n* = 65)	*P*-value	2023 Q2 (*n* = 136)	2024 Q2 (*n* = 94)	*P*-value	2023 Q3 (*n* = 132)	2024 Q3 (*n* = 100)	*P*-value
Age, years	66 (SD: 13)	66 (SD: 14)	.876	67 (SD: 11)	67 (SD: 12)	.892	66 (SD: 15)	66 (SD: 11)	.866
Sex, male	106 (68.8)	49 (75.4)	.330	91 (66.4)	69 (73.4)	.259	98 (74.2)	74 (74.0)	.967
Body mass index, kg/m^2^	25 (SD: 4)	24 (SD: 3)	.105	25 (SD: 4)	24 (SD: 3)	.317	25 (SD: 4)	25 (SD: 4)	.331
Hypertension	114 (74.0)	50 (76.9)	.734	101 (73.7)	66 (70.2)	.558	85 (64.4)	69 (69.0)	.462
Diabetes mellitus	48 (31.2)	22 (33.8)	.698	54 (39.4)	25 (26.6)	.044	49 (37.1)	35 (35.0)	.739
Cerebrovascular accident	27 (17.5)	13 (20.0)	.666	22 (16.1)	12 (12.8)	.488	21 (15.9)	16 (16.0)	.985
Chronic kidney disease	20 (13.0)	14 (21.5)	.110	19 (14.0)	10 (10.6)	.454	19 (14.4)	11 (11.0)	.445
Medical aid	6 (3.9)	1 (1.5)	.677	6 (4.4)	1 (1.1)	.245	8 (6.1)	4 (4.0)	.483
Albumin, g/dl	3.9 (SD: 0.5)	3.9 (SD: 0.6)	.474	4.0 (SD: 0.5)	4.0 (SD: 0.5)	.635	3.9 (SD: 0.6)	4.1 (SD: 0.5)	.004
EuroSCORE II	1.9 [1-5]	4.0 [2-8]	<.001	2.0 [1-4]	2.2 [1-5]	.058	2.2 [1-5]	2.0 [1-3]	.549
LV dysfunction	16 (10.7)	6 (9.5)	.802	10 (7.5)	8 (8.6)	.755	9 (7.0)	9 (9.0)	.569
Coronary, *n*	68	13	.533	47	23	.682	31	30	.624
1-vessel disease	1 (1.5)	0 (0)		1 (2.1)	0 (0)		1 (3.2)	2 (6.7)	
2-vessel disease	14 (20.6)	1 (7.7)		8 (17.0)	6 (26.1)		7 (22.6)	4 (13.3)	
3-vessel disease	53 (77.9)	12 (92.3)		38 (80.9)	17 (73.9)		23 (74.2)	24 (80.0)	
Valve, *n*	34	10	.783	45	27	.747	45	26	.591
Single valve surgery	20 (58.8)	7 (70.0)		37 (82.2)	24 (88.9)		37 (82.2)	19 (73.1)	
Double valve surgery	13 (38.2)	3 (30.0)		6 (13.3)	3 (11.1)		7 (15.6)	6 (23.1)	
Triple valve surgery	1 (2.9)	0 (0)		2 (4.4)	0 (0)		1 (2.2)	1 (3.8)	
Infective endocarditis	3 (8.8)	3 (33.3)	.120	3 (6.7)	1 (3.7)	>.999	4 (8.9)	3 (11.5)	.701
Aorta, *n*	52	42		44	44		56	44	
Aneurysm	29 (55.8)	20 (47.6)	.432	23 (52.3)	31 (68.9)	.109	30 (53.6)	27 (62.8)	.358
Acute aortic syndrome	18 (34.6)	13 (41.9)	.707	15 (34.1)	6 (13.3)	.021	22 (39.3)	7 (16.3)	.013
Complex surgery	44 (28.6)	10 (15.4)	.039	40 (29.2)	22 (23.4)	.329	33 (25.0)	17 (17.0)	.142
Redo OHS	12 (7.8)	9 (13.8)	.165	9 (6.6)	12 (12.8)	.112	14 (10.6)	10 (10.0)	.881
Emergency surgery	23 (14.9)	18 (27.7)	.027	18 (13.1)	7 (7.4)	.171	22 (16.7)	14 (14.0)	.579
Operation time, min	260 [220-310]	260 [225-325]	.974	270 [225-315]	228 [190-275]	<.001	265 [230-338]	235 [185-285]	<.001

Data are presented as number (%) for categorical variables and as the mean (SD) or median [interquartile range] for continuous variables.

Abbreviation: LV dysfunction: preoperative left ventricular ejection fraction <40%.

The EuroSCORE II was significantly higher in 2024 Q1 compared with 2023 Q1 (2023/2024 Q1: median 1.9 [IQR 1-5] vs 4.0 [2-8], *P* < .001). The proportion of acute aortic syndrome was lower in both 2024 Q2 and Q3 compared with the corresponding periods in 2023. In 2023 Q2, acute aortic syndrome occurred in 15 patients (34.1%) compared with 6 patients (13.3%) in 2024 Q2 (*P* = .021). Similarly, in 2023 Q3, the proportion was 22 patients (39.3%) compared with 7 patients (16.3%) in 2024 Q3 (*P* = .013). Complex surgery was less frequent in 2024 Q1 than in 2023 Q1 (2023/2024 Q1: 28.6% vs 15.4%, *P* = .039), whereas emergency surgery was more frequent in 2024 Q1 than in 2023 Q1 (2023/2024 Q1: 14.9% vs 27.7%, *P* = .027). Operation time was significantly shorter in 2024 Q2 and Q3 compared with the corresponding quarters in 2023 (2023/2024 Q2: 270 [225-315] vs 228 [190-275] min, *P* < .001; 2023/2024 Q3: 265 [230-338] vs 235 [185-285] min, *P* < .001).

### Surgery-related outcomes

The surgery-related outcomes are summarized in **Table 2**, **Figure 2B and C**. Red blood cell (RBC) transfusion was higher in 2024 Q1 than in 2023 Q1 (2023/2024 Q1: 4 [1-8] vs 6 [3-8] units, *P* = .044), whereas it was lower in 2024 Q3 compared with 2023 Q3 (2023/2024 Q3: 4 [2-8] vs 3 [0-5] units, *P* < .001). Ventilator duration was significantly shorter in 2024 Q2 and Q3 than in the corresponding quarters of 2023 (2023/2024 Q2: 7 [4-15] vs 5 [3-11] h, *P* = .009; 2023/2024 Q3: 8 [4-16] vs 4 [3-8] h, *P* < .001). Similarly, ICU stay was shorter in 2024 Q2 and Q3 (2023/2024 Q2: 50 [38-74] vs 26 [21-50] h, *P* < .001; 2023/2024 Q3: 67 [28-99] vs 27 [23-48] h, *P* < .001). Hospital stay was longer in 2024 Q1 than in 2023 Q1 (2023/2024 Q1: 9 [7-14] vs 11 [8-19] days, *P* = .012) (**Figure 2B**).

**Table 2.** Comparisons of Postoperative Outcomes Between 2023 and 2024 Groups

	2023 Q1 (*n* = 154)	2024 Q1 (*n* = 65)	*P*-value	2023 Q2 (*n* = 136)	2024 Q2 (*n* = 94)	*P*-value	2023 Q3 (*n* = 132)	2024 Q3 (*n* = 100)	*P*-value
Bleeding control surgery	18 (11.7)	5 (7.7)	.474	10 (7.4)	6 (6.4)	.801	10 (7.4)	6 (6.0)	.497
Reoperation	9 (5.8)	6 (9.2)	.387	11 (8.1)	5 (5.3)	.447	11 (8.3)	6 (6.0)	.614
Stroke	7 (4.5)	1 (1.5)	.441	2 (1.5)	4 (4.3)	.229	2 (1.5)	4 (4.0)	.407
Respiratory complication	30 (19.5)	17 (26.2)	.272	18 (13.2)	20 (21.3)	.106	27 (20.5)	14 (14.0)	.202
Acute renal failure	8 (5.2)	6 (9.2)	.363	9 (6.6)	3 (3.2)	.368	8 (6.1)	7 (7.0)	.793
Life-threatening arrhythmia	8 (5.2)	2 (3.1)	.727	5 (3.7)	6 (6.4)	.363	5 (3.8)	4 (4.0)	>.999
Postoperative MI	3 (1.9)	1 (1.5)	>.999	1 (0.7)	1 (1.1)	>.999	1 (0.8)	2 (2.0)	.579
Culture-positive sepsis	0 (0)	1 (1.5)	.297	2 (1.5)	3 (3.2)	.401	2 (1.5)	2 (2.0)	>.999
Wound complication	17 (11.0)	5 (7.7)	.480	9 (6.6)	9 (9.6)	.459	6 (4.5)	7 (7.0)	.566
Mediastinitis	3 (1.9)	0 (0)	.557	2 (1.5)	3 (3.2)	.401	1 (0.8)	3 (3.0)	.318
Readmission	12 (7.8)	8 (12.3)	.310	8 (5.9)	4 (4.3)	.766	11 (8.3)	11 (11.0)	.506
30-day mortality	6 (3.9)	3 (4.6)	.727	4 (2.9)	3 (3.2)	>.999	3 (2.3)	5 (5.0)	.295
FTR complication	54 (35.1)	27 (41.5)	.444	37 (27.2)	34 (36.2)	.191	46 (34.8)	32 (32.0)	.676
FTR	6 (11.1)	6 (22.2)	.201	4 (10.8)	6 (17.6)	.504	4 (8.7)	6 (18.8)	.302
RBC transfusion, units	4 [1-8]	6 [3-8]	.044	4 [1-7]	4 [2-7]	.637	4 [2-8]	3 [0-5]	<.001
Ventilator use, h	8 [5-18]	10 [6-21]	.433	7 [4-15]	5 [3-11]	.009	8 [4-16]	4 [3-8]	<.001
Intensive care unit stay, h	44 [25-74]	44 [25-70]	.909	50 [38-74]	26 [21-50]	<.001	67 [28-99]	27 [23-48]	<.001
Hospital stay, days	9 [7-14]	11 [8-19]	.012	9 [7-13]	10 [8-17]	.087	9 [7-15]	9 [7-15]	.759
Surgery waiting time, days	14 [8-22]	44 [21-71]	<.001	17 [10-27]	37 [22-55]	<.001	27 [11-43]	29 [15-55]	.056
Chest tube drainage, h	103 [68-162]	118 [82-205]	.102	113 [71-162]	101 [70-187]	.557	138 [93-208]	98 [67-162]	<.001
Vasopressors/inotropes, h	52 [30-95]	60 [28-110]	.686	67 [39-110]	82 [23-140]	.340	80 [47-125]	61 [26-134]	.118

Data are presented as number (%) for categorical variables and the median [interquartile range] for continuous variables.

Abbreviations: FTR: failure-to-rescue; MI: myocardial infarction; RBC: red blood cell.

Surgical waiting time was significantly prolonged in both 2024 Q1 and Q2 compared with the corresponding periods in 2023 (2023/2024 Q1: 14 [8-22] vs 44 [21-71] days, *P* < .001; 2023/2024 Q2: 17 [10-27] vs 37 [22-55] days, *P* < .001) (**Figure 2C**). Chest tube duration was shorter in 2024 Q3 than in 2023 Q3 (2023/2024 Q3: 138 [93-208] vs 98 [67-162] h, *P* < .001).

### Cases of aortic emergencies

Among 181 emergency aortic cases, 106 occurred in 2023, and 75 occurred in 2024 representing a 29.2% reduction in case volume in 2024 (**Table 3**). Most clinical features were similar. However, transfers from other tertiary hospitals were significantly more common in 2024 than in 2023 (20.3% vs 8.4%, *P* = .040).

**Table 3.** Baseline Characteristics and Clinical Outcomes of Emergency Aortic Cases

	2023 (*n* = 106)	2024 (*n* = 75)	*P*-value
Sex, male	77 (73.3)	47 (62.7)	.144
Age	66 (SD: 15)	65 (SD: 13)	.498
Admission route			.975
Emergency room	21 (19.8)	15 (20.0)	
Transfer	85 (80.2)	60 (80.0)	
Treatment option			.299
Medical	37 (34.9)	25 (33.3)	
Intervention	2 (1.9)	5 (6.7)	
Surgery	67 (63.2)	45 (60.0)	
Acute type A aortic dissection	56 (52.8)	30 (40.0)	.089
Acute type B aortic dissection	25 (23.6)	27 (36.0)	.069
Aortic aneurysm	25 (23.6)	18 (24.0)	.948
30-day mortality	4 (4.2)	6 (8.6)	.325
Symptom-to-hospital, h	6 [4-11]	6 [4-18]	.502
Transfer from tertiary hospital	7 (8.4)	12 (20.3)	.040

Data are presented as number (%) for categorical variables and the median [interquartile range] for continuous variables.

Abbreviation: Symptom-to-hospital: interval between symptom onset and arrival at our institution.

### Risk factor analyses

#### Thirty-day mortality

On multivariable logistic regression analyses, independent predictors were valve surgery (OR 15.49, 95% CI 2.08-115.52; *P* = .008), diabetes mellitus (OR 3.33, 95% CI 1.11-9.98; *P* = .032), preoperative left ventricular dysfunction (OR 9.02, 95% CI 2.47-32.89; *P* < .001), and greater RBC transfusion (OR 1.44, 95% CI 1.28-1.62; *P* < .001). Resident absence was not a significant independent predictor of 30-day mortality (OR 1.62, 95% CI 0.56-4.69; *P* = .374) (**Supplementary Material 4**).

#### FTR complications

**Figure 3.**
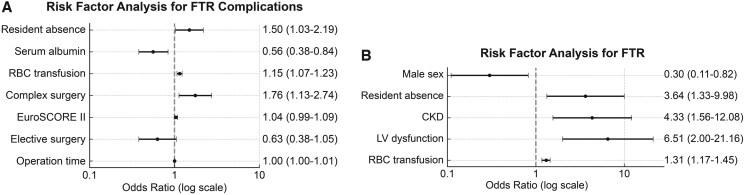
Forest Plots of Multivariable Logistic Regression Analyses for the Risk of Failure-to-Rescue (FTR) Complications and FTR. (A) Risk factor analysis of FTR complications, and (B) risk factor analysis of FTR. Abbreviations: CKD: chronic kidney disease; FTR: failure-to-rescue; LV: left ventricle; RBC: red blood cell.

On multivariable logistic regression analyses, independent risk factors included resident absence (OR 1.50, 95% CI 1.03-2.19; *P* = .034), greater RBC transfusion (OR 1.15, 95% CI 1.07-1.23; *P* < .001), complex surgery (OR 1.76, 95% CI 1.13-2.74; *P* = .012), and longer operation time (OR 1.004, 95% CI 1.002-1.006; *P* < .001), whereas a higher preoperative serum albumin level was a protective factor (OR 0.56, 95% CI 0.38-0.84; *P* = .005) (**Figure 3A** and **Supplementary Material 5**).

##### Failure-to-rescue

On multivariable logistic regression analyses, independent risk factors included resident absence (OR 3.64, 95% CI 1.33-9.98; *P* = .012), chronic kidney disease (OR 4.33, 95% CI 1.56-12.08; *P* = .005), preoperative left ventricular dysfunction (OR 6.51, 95% CI 2.00-21.16; *P* = .002), and greater RBC transfusion (OR 1.31, 95% CI 1.17-1.45; *P* < .001). Male sex was a protective factor (OR 0.30, 95% CI 0.11-0.82; *P* = .018) (**Figure 3B** and **Supplementary Material 6**).

### Medical staff workload

The workloads of PAs, SAs, and consultants are shown in **Figure 2D**, and detailed data by quarter are provided in **Supplementary Material 7**. The weekly working hours of SAs and consultants were significantly longer in 2024 than in 2023 (SA: 46 [44-48] vs 50 [48-53] h, *P* < .001; consultants: 60 [60-60] vs 80 [60-85] h, *P* < .001).

## DISCUSSION

The main findings of this study are as follows:

1. The surgical volume decreased sharply early after resident absence, with higher EuroSCORE II and emergency rates. These changes likely reflected the prioritization of emergencies.
2. The operation time, duration of ventilator support, and length of ICU stay decreased over time, whereas the complication rates and length of hospital stay remained unchanged. This finding indicates that improvements in operating room and ICU did not directly translate into better overall clinical outcomes.
3. Thirty-day mortality did not change, and resident absence was not an independent risk factor.
4. Resident absence independently increased FTR complications and FTR risk. This finding suggests that patients who might have survived with resident support were more likely to die.
5. Transfers between tertiary centres increased, suggesting a structural vulnerability in the healthcare delivery system.

### Changes in clinical practice

What had previously been conceivable only as a hypothetical scenario—the nationwide disappearance of residents—became reality in South Korea.

The first major change was the postponement of elective surgeries and a shift towards emergencies, with higher EuroSCORE II. This shift likely reflected the prioritization of limited workforce for life-saving care and a shortage of anaesthesiologists.

Operation time decreased, likely because surgeons no longer needed to spend time on education in the operating room. However, this alteration does not reflect changes in surgery type or complexity and should be interpreted with caution. In 2024 Q3, the durations of ventilation, ICU stay decreased, possibly because of faster decision-making by consultants and reduced surgical volume, which facilitated timely ICU-to-ward transfers. However, complication rates and hospital stay duration did not change, likely because other variables may have offset this effect, and patients could be discharged without time pressure, allowing longer recovery in the ward.

Surgical waiting times were prolonged throughout the period of resident absence. As noted in previous studies, the impact of surgical delay is difficult to predict and may pose long-term risks, highlighting the need for long-term follow-up.

### Mortality and failure-to-rescue

The year of surgery was used as a surrogate because CV surgery policy remained stable. Although unmeasured factors may exist, they were regarded as part of the natural adaptation to residents’ resignation.

Failure-to-rescue, defined as death after a postoperative complication, was first introduced by Silber et al^7^ and has since been validated as a quality metric for postoperative care in numerous studies.^8–10^ FTR complications vary by surgery type, but the inclusion criteria should follow certain principles: (1) severe enough to potentially result in death, (2) not present preoperatively, and (3) potentially preventable or amenable to rescue.^11^

Consistent with the findings of previous studies, 30-day mortality did not increase, and resident absence was not an independent risk factor.^6^^,^^12^^,^^13^ Although the crude comparison of FTR complications and FTR showed no significant differences when comparing by quarter, but after controlling for confounding variables in the multivariable analysis, the resident absence independently predicted FTR complications and FTR, suggesting possibility of failures in early detection and timely intervention. Providing standardized care protocols and replacing residents with other medical staff may have been insufficient to compensate for resident absence.

Other independent risk factors for FTR were male, chronic kidney disease, preoperative left ventricular dysfunction, and greater RBC transfusion, which were generally consistent with previous reports.^14^^,^^15^

Possible explanations may account for our less favourable results compared with previous studies. CV surgery involves high-risk patients who require close monitoring and immediate intervention. According to the Korean Intern Resident Association Survey 2022, CV surgery residents worked an average of 102 h weekly, indicating the system’s high dependency on their labour. Moreover, most previous studies have assessed short-term absences and may not reflect the impact of prolonged absences. Finally, negative outcomes may have been underreported, particularly in single-centre studies.

### Emergency healthcare delivery system

The increase in transfers between tertiary centres is undesirable and reveals structural vulnerabilities in the healthcare delivery system, as longer travel may adversely affect outcomes.^16^ It is concerning that tertiary hospitals, which should serve as the final destination for emergency patients, failed to fulfil this role. The lower proportion of acute type A dissections and unchanged symptom-to-hospital arrival time may suggest the presence of survivor bias in 2024. Further studies with nationwide data are needed for validation.

### Financial and workload changes

In 2024 Q1, revenue dropped to 53% of 2023 Q1 levels and has not returned to baseline (**Figure 2A**). The workloads of SAs and consultants increased, raising concerns about burnout and financial sustainability (**Figure 2D**), as their hourly wages are substantially higher than those of residents. These increased costs were covered by public funds based on taxation (**Supplementary Material 3**).

### Limitations

First, the single-centre, retrospective design and the relatively small number of FTR events relative to the number of covariates may have limited the statistical power of our analyses. Second, since reliance on residents varies internationally, these findings may not be generalizable. Further studies with nationwide data are needed for validation. Nevertheless, discrepancy between documented working hours and actual working hours is not unique to South Korea and has been reported in resident training systems in other countries as well. For this reason, we believe our observations in the setting of resident overwork may provide meaningful insights for the healthcare system.

## CONCLUSION

The nationwide mass resignation of residents revealed the structural vulnerability of South Korea’s healthcare system, which has relied heavily on residents. In their absence, teaching hospitals experienced difficulties in maintaining prior surgical volumes, raising concerns about possible downstream effects on postoperative care quality and the healthcare delivery system. The remaining staff appeared to face heavier workloads, and patients experienced longer waiting times for surgery, which may threaten the sustainability of the healthcare system. These findings suggest that careful reform of this resident-dependent system may be warranted, and teaching hospitals should continue to emphasize their role as educational institutions, ensuring that residents are regarded primarily as trainees rather than as a low-cost workforce.

## Supplementary Material

ivag037_Supplementary_Data

## Data Availability

Due to restrictions by our institutional ethics committee, raw data cannot be shared.
