# Supplementary material for: Impact of Residents’ Mass Resignation in Cardiovascular Surgery: A System Sustainability Perspective
Source: Interdiscip Cardiovasc Thorac Surg. 2026 Feb 6;41(2):ivag037. doi: 10.1093/icvts/ivag037 (PMC12915579; doi:10.1093/icvts/ivag037)
Supplement: ivag037_Supplementary_Data [file ivag037_supplementary_data.docx]

**Supplementary Material for the Manuscript**

**“Impact of Residents’ Mass Resignation in Cardiovascular Surgery: A System Sustainability Perspective”**

June Yeop Lee^1,2^, Hyoung Woo Chang^1,2*^, Sang Yoon Kim^1,2^, Joon Chul Jung^1,2^, Jae Hang Lee^1,2^, Sanghon Park^1,2^, Jun Sung Kim^1,2^, Kay-Hyun Park^1,2^

^1^Department of Thoracic and Cardiovascular Surgery, Seoul National University Bundang Hospital, Bundang-gu, Seongnam-si, Gyeonggi-do 13620, Republic of Korea

^2^Department of Thoracic and Cardiovascular Surgery, Seoul National University College of Medicine, Jongno-gu, Seoul 03080, Republic of Korea

**Table of contents**

**Supplementary Material 1. Definitions of postoperative complications and outcomes**

**Supplementary Material 2. Timeline of nationwide resident resignation in South Korea**

**Supplementary Material 3. Administrative and financial changes**

**Supplementary Material 4. Risk factor analysis for 30-day mortality**

**Supplementary Material 5. Risk factor analysis for failure-to-rescue (FTR) complications**

**Supplementary Material 6. Risk factor analysis for failure-to-rescue (FTR)**

**Supplementary Material 7. Number of staff and weekly working hours by occupation**

**Supplementary Material 8. End of the resident resignation**

**Supplementary Material 1.** **Definitions of postoperative complications and outcomes**

| Complications | Definitions |
| --- | --- |
| Bleeding | Patient returned to the operating room for mediastinal bleeding or cardiac tamponade |
| Reoperation | Patient returned to the operating room for any reason except for bleeding or cardiac tamponade |
| Stroke | New-onset ischaemic or haemorrhagic stroke diagnosed by computed tomography or magnetic resonance imaging |
| Respiratory | Prolonged mechanical ventilation (>24 hours), pneumonia or tracheostomy |
| Acute renal failure | An increase in serum creatinine (Cr) ≥ 3.0 times the baseline or increase of serum Cr > 4.0 (mg/mL) or reduction in urine output < 0.3 mL/kg/hour over 12 hours or new requirement for dialysis |
| Life-threatening arrhythmia | Arrhythmias that can cause sudden cardiac arrest, haemodynamic collapse, or death without intervention (for example. sustained ventricular tachycardia, ventricular fibrillation, complete AV block requiring pacemaker insertion) |
| Wound | Wound complication including dehiscence, consistent oozing or superficial infection |
| Mediastinitis | Deep sternal wound infection characterized by positive results of computed tomography and/or culture, necessitating re-exploration |
| Culture-positive sepsis | An increase of 2 points or more from the baseline sequential organ failure assessment (SOFA) score in the presence of infection. Because clinical suspicion of infection can be subjective, in this study, sepsis was defined only when a pathogen was clearly identified in culture results |
| Readmission | Hospital readmission due to surgery-related complications within 30 days after discharge |
| Thirty-day mortality | Death occurring within 30 days post-operatively, regardless of inside or outside the hospital |
| Hospital mortality | All-cause death occurring during hospital admission |
| Failure-to-rescue (FTR) | Hospital mortality in any patient who had one or more of the following complications: postoperative acute renal failure, prolonged mechanical ventilation > 24 hours, stroke and reoperation including bleeding control surgery, life-threatening arrhythmia, postoperative MI, and sepsis |
| Surgical waiting time | The duration between the date of surgical decision and the date of surgery in elective cases. |

Cr; creatinine, MI; myocardial infarction

**Supplementary Material 2. Timeline of nationwide resident resignation in South Korea (Feb, 1, 2024–Feb, 28, 2025)**

| Timeline | Events |
| --- | --- |
| Feb 6, 2024 | The Minister of Health and Welfare chaired a meeting of the Health and Medical Policy Deliberation Committee and announced the decision to increase the medical school admission quota from 3,058 to 5,058 starting with the current recruitment year. |
| Feb 7, 2024 | Anticipating collective action by doctors and medical students led by the Korean Medical Association (KMA), the Ministry of Health and Welfare preemptively issued an order to the KMA prohibiting collective action. |
| Feb 19, 2024 | Most medical students submitted leave-of-absence requests, and over 95% of residents at 221 teaching hospitals nationwide submitted resignation letters. Residents chose resignation over striking because strikes by medical personnel are not legally permitted in South Korea. |
| Feb 20, 2024 | Medical students and residents took leave and resigned. The Ministry of Education banned universities from approving leaves of absence, warning of penalties in future student recruitment. The Ministry of Health and Welfare ordered hospitals to maintain services, reject resignations, and prevent residents from working elsewhere. As residents can be affiliated with only one institution under Korean law, they could not work at other hospitals until these orders were lifted and teaching hospitals processed the resignation letters on July 15, 2024. |
| Feb 23, 2024 | Due to severe disruptions in patient care at teaching hospitals—primarily tertiary hospitals—caused by the mass resignation of residents, the Ministry of Health and Welfare raised the public health disaster alert level to the highest of four stages, ‘Severe’. |
| Feb 27, 2024 | The Ministry of Health and Welfare launched a ‘Pilot Project on Expanding Nursing Roles’, broadening the scope of 98 procedures previously restricted to physicians. These included medical record documentation, performing endotracheal intubation and extubation, inserting central venous catheters, removing drainage tubes, and suturing. |
| Mar 3, 2024 | The World Medical Association issued a statement supporting Korean doctors' actions and urging changes in the government's response. |
| Mar 5, 2024 | The Ministry of Health and Welfare issued preliminary administrative notices of a three-month suspension of medical licences to residents who had submitted resignations. This was possible because the government has authority over medical licensure in South Korea. |
| Mar 11, 2024 | The government conducted the first round of dispatches, sending public health physicians and military physicians from regional health centres and military units to teaching hospitals. |
| Mar 13, 2024 | The Korean Intern Resident Association requested urgent intervention from the International Labour Organization (ILO), arguing that the government’s orders banning resignation acceptance and forcing work violated constitutional rights, including freedom of occupation, professional practice, and the pursuit of happiness. |
| Mar 16–20, 2024 | The Ministry of Education held the 1st, 2nd, and 3rd meetings of the 'Medical School Quota Allocation Review Committee' to finalize quota allocations for each university. Participant lists and meeting records were not disclosed and were destroyed by the Ministry. |
| Mar 18, 2024 | The Ministry of Health and Welfare suspended the medical licences of two KMA executives for three months on charges of inciting resident action. |
| Mar 20, 2024 | The Ministry of Education announced the 2025 medical school admission quota allocations by university. Regional medical schools outside Seoul, the capital of South Korea, saw increases ranging from 7.5% to 308%. |
| Mar 25, 2024 | Medical school professors nationwide submitted resignation letters in protest of government policy, although the resignations were not enacted. |
| Mar 29, 2024 | The ILO decided to proceed with an intervention, sending letters to both the government and the resident association. The Ministry of Employment and Labour argued that residents are not part of a labour union and therefore are not protected under ILO labour conventions. |
| Apr 10, 2024 | General elections for the National Assembly were held. |
| Apr 19, 2024 | The Ministry of Health and Welfare allowed university presidents to admit between 50% and 100% of their allocated medical school quota at their discretion. |
| Apr 25, 2024 | A Presidential Commission on Medical Reform was launched without participation from the KMA or the resident association. Chaired by a former pharmaceutical executive, the 27-member body included only three hospital directors from the medical community, with the rest consisting of government officials, civic organization leaders, dentists, nurses, traditional medicine doctors, and pharmacists, etc. |
| May 3, 2024 | The resident association filed an administrative lawsuit against the Ministry of Health and Welfare, alleging abuse of authority and violation of constitutional rights by issuing orders to maintain medical services, prohibit resignation acceptance, and compel work. The case is ongoing. |
| May 16, 2024 | The Seoul High Court dismissed medical students’ request for an injunction to halt the quota expansion. The ruling acknowledged insufficient evidence for the increase and concerns about infringement on the right to education, but prioritized public interest. |
| May 24, 2024 | The Korean Council for University Education, an association composed of university presidents, reviewed and approved the 2025 university admissions implementation plan. |
| Jun 4, 2024 | The Ministry of Health and Welfare rescinded orders to maintain medical services, prohibit resignation acceptance, and compel work. |
| Jul 10, 2024 | The Ministry of Education issued ‘Guidelines for Flexible Operation of Medical School Curricula’. Since leaves of absence were prohibited, medical students staged class boycotts. The guidelines allowed fewer class days, remote learning, and the use of ‘I’ (incomplete) grades instead of failing grades to avoid academic failure. As leaves were not approved, tuition refunds were not granted. |
| Jul 15, 2024 | Teaching hospitals processed the resignation letters submitted by residents in February 20, after which many residents found employment in private clinics or hospitals. Civil lawsuits are ongoing over financial losses incurred during the prohibition on accepting resignations. |
| Aug 21–Sep 13, 2024 | The Seoul Metropolitan Police Agency summoned six resident representatives from major teaching hospitals in Seoul for questioning. |
| Aug 28, 2024 | The National Assembly passed the Nursing Act, expanding the scope of nursing practice. |
| Dec 3, 2024 | President Yoon declared martial law, with the proclamation stating that residents who did not return within 48 hours would be cracked down on under martial law. This triggered renewed backlash from the medical community. President Yoon has since been impeached and is now on trial for insurrection. |
| Dec 4-9, 2024 | A total of 181 new residents were recruited nationwide for 2025, representing 5.0% of the recruitment quota of 3,594. |
| Dec 12, 2024 | The 'Doctors for a Fair Society' group filed a lawsuit for damages against the Korea Institute for Health and Social Affairs, one of three government-cited reports justifying the quota increase, alleging calculation errors and intentional bias. The court acknowledged some typographical and calculation errors but ruled that intentional manipulation was not proven. |
| Jan, 23, 2025 | The 2025 national medical licensing exam produced 269 new physicians, 8.8% of the 3,045 new physicians in 2024. The pass rate fell from 94.2% in 2024 to 70.4% in 2025. |
| Jan, 31, 2025 | A total of 1,171 of 13,531 training positions (8.7%) were filled: interns, 100 of 3,058 (3.3%); residents, 1,071 of 10,463 (10.2%). In national cardiothoracic surgery, only six residents obtained board certification in 2025, compared with 32 in 2022, 32 in 2023, and 21 in 2024. |
| Feb, 12, 2025 | The Korean Institute of Medical Education and Evaluation denied accreditation to three of 30 medical schools whose quotas had increased by more than 10%. If these schools fail re-evaluation in one year, their student recruitment will be suspended. The institute is recognized by the World Federation for Medical Education (WFME) as an accrediting body. |

**Supplementary Material 3. Administrative and financial changes**

***Changes in administrative measures after residents’ mass resignation***

The government’s first administrative measure was to issue an executive order prohibiting teaching hospitals from accepting residents’ resignations and to send advance notice of licence suspension to residents who had submitted resignation letters. The Ministry of Health and Welfare ordered hospitals to reject resignations and prevent residents from working elsewhere. As residents can be affiliated with only one institution under Korean law, they could not work at other hospitals until these orders were lifted on June 4, 2024.

The government also redeployed public health physicians and military physicians from regional health centres and military units to major teaching hospitals to substitute for residents’ work. These were male physicians who, under the Military Service Act, had been serving 38-month terms in remote islands or rural areas, contributing to the reduction in regional healthcare disparities and to military medicine. Consequently, their reassignment may have negatively affected the quality of care in medically underserved areas. In our institution, one board-certified cardiovascular surgeon serving as a military physician was dispatched for one month in August 2024 to work on the general ward.

As faculty members assumed the responsibilities previously delegated to residents, numerous consultants resigned because of burnout. Teaching hospitals began notifying the Emergency Medical Control Center that they could not accept emergency patients because of staff shortages. In response, the government issued an order stipulating that emergency patients must be accepted regardless of staffing levels, with penalties for noncompliance. This order was later withdrawn because of strong opposition from frontline clinicians. The government subsequently adopted a strategy of providing financial support from the National Health Insurance budget and local government disaster management funds to substantially increase on-call pay for specialists.

Nevertheless, the situation persists to this day, with many consultants, particularly in regional teaching hospitals, resigning to open private clinics or transfer to private hospitals.

***Financial measures and changes after residents’ mass resignation***

As part of its financial support measures for teaching hospitals, the South Korean government allocated substantial financial resources (€2.3 billion, on February 5, 2025). This included €141 million from the reserve fund, €151 million from local government disaster management funds, €1.0 billion from emergency medical system funds, and €1.0 billion in NHIS advance payments to teaching hospitals. These funds were used for on-call allowances, salaries for newly hired healthcare staff, and allowances for dispatched military and public health physicians. This amount accounted for 25.6% of the €7.8 billion deficit recorded by the NHIS in one year, accelerating the depletion of NHIS finances, with additional funding still being provided.

**Supplementary Material 4. Risk factor analysis for 30-day mortality.** The results of univariable and multivariable logistic regression analyses. Odds ratios with 95% confidence intervals and corresponding p values are presented

| **30-day mortality** | **Univariable analysis** | | **Multivariable analysis** | |
| --- | --- | --- | --- | --- |
|  | **OR (95% CI)** | **P value** | **OR (95% CI)** | **P value** |
| Surgery type |  |  |  |  |
| Coronary |  |  |  |  |
| **Valve** | **3.25 (1.02-10.39)** | **0.047** | **15.49 (2.08-115.52)** | **0.008** |
| Aorta | 1.71 (0.52-5.64) | 0.375 | 3.22 (0.40-25.99) | 0.272 |
| **Male sex** | **0.32 (0.14-0.73)** | **0.007** |  |  |
| **Age** | **1.07 (1.02-1.12)** | **0.004** |  |  |
| **Resident absence** | **1.40 (0.62-3.16)** | **0.425** | **1.62 (0.56-4.69)** | **0.374** |
| Medical aid | 2.40 (0.53-10.79) | 0.254 |  |  |
| Body mass index (kg/m^2^) |  |  |  |  |
| Normal (ref)  (≥18.5, <25) |  |  |  |  |
| **Underweight  (<18.5)** | **3.35 (0.90-12.53)** | **0.072** |  |  |
| **Overweight  (≥25)** | **0.44 (0.17-1.16)** | **0.097** |  |  |
| Hypertension | 1.56 (0.57-4.23) | 0.385 |  |  |
| **Diabetes mellitus** | **1.99 (0.88-4.49)** | **0.099** | **3.33 (1.11-9.98)** | **0.032** |
| Cerebrovascular accident | 1.37 (0.50-3.74) | 0.542 |  |  |
| **Chronic kidney disease** | **5.01 (2.15-11.65)** | **<0.001** | **2.80 (0.96-8.15)** | **0.059** |
| **Preoperative albumin** | **0.18 (0.09-0.34)** | **<0.001** |  |  |
| **LV dysfunction** | **9.06 (3.82-21.48)** | **<0.001** | **9.02 (2.47-32.89)** | **<0.001** |
| Redo surgery | 0.40 (0.05-2.98) | 0.368 |  |  |
| **RBC transfusion** | **1.38 (1.26-1.51)** | **<0.001** | **1.44 (1.28-1.62)** | **<0.001** |
| **Complex surgery** | **2.58 (1.10-6.03)** | **0.029** |  |  |
| **EuroSCORE II** | **1.14 (1.09-1.19)** | **<0.001** |  |  |
| **Elective surgery** | **0.35 (0.15-0.81)** | **0.015** |  |  |
| **Operation time** | **1.005 (1.002-1.008)** | **<0.001** |  |  |

Abbreviations: OR = odds ratio; CI = confidence interval; LV dysfunction = preoperative left ventricular dysfunction (ejection fraction < 40%).

**Supplementary Material 5. Risk factor analysis for failure-to-rescue (FTR) complications.** The results of univariable and multivariable logistic regression analyses. Odds ratios with 95% confidence intervals and corresponding p values are presented

| **FTR Complication** | **Univariable analysis** | | **Multivariable analysis** | |
| --- | --- | --- | --- | --- |
|  | **OR (95% CI)** | **P value** | **OR (95% CI)** | **P value** |
| Surgery type |  |  |  |  |
| Coronary |  |  |  |  |
| **Valve** | **1.72 (1.12-2.66)** | **0.014** |  |  |
| **Aorta** | **2.11 (1.42-3.13)** | **<0.001** |  |  |
| Male sex | 0.95 (0.67-1.35) | 0.791 |  |  |
| **Age** | **1.01 (0.99-1.02)** | **0.105** |  |  |
| **Resident absence** | **1.17 (0.84-1.61)** | **0.357** | **1.50 (1.03-2.19)** | **0.034** |
| **Medical aid** | **2.02 (0.92-4.43)** | **0.080** |  |  |
| Body mass index (kg/m^2^) |  |  |  |  |
| Normal  (≥18.5, <25) |  |  |  |  |
| **Underweight (<18.5)** | **3.73 (1.54-9.06)** | **0.004** |  |  |
| Overweight (≥25) | 0.93 (0.67-1.29) | 0.660 |  |  |
| Hypertension | 1.15 (0.80-1.63) | 0.453 |  |  |
| Diabetes mellitus | 1.11 (0.80-1.55) | 0.533 |  |  |
| Cerebrovascular accident | 1.24 (0.81-1.89) | 0.323 |  |  |
| **Chronic kidney disease** | **2.32 (1.49-3.61)** | **<0.001** |  |  |
| **Preoperative albumin** | **0.33 (0.24-0.46)** | **<0.001** | **0.56 (0.38-0.84)** | **0.005** |
| **LV dysfunction** | **1.80 (1.05-3.10)** | **0.033** |  |  |
| Redo surgery | 1.05 (0.62-1.80) | 0.846 |  |  |
| **RBC transfusion** | **1.29 (1.22-1.36)** | **<0.001** | **1.15 (1.07-1.23)** | **<0.001** |
| **Complex surgery** | **2.13 (1.45-3.14)** | **<0.001** | **1.76 (1.13-2.74)** | **0.012** |
| **EuroSCORE II** | **1.15 (1.11-1.20)** | **<0.001** | **1.04 (0.99-1.09)** | **0.088** |
| **Elective surgery** | **0.32 (0.22-0.48)** | **<0.001** | **0.63 (0.38-1.05)** | **0.062** |
| **Operation time** | **1.006 (1.004-1.008)** | **<0.001** | **1.004 (1.002-1.006)** | **<0.001** |

Abbreviations: FTR; failure-to-rescue, OR; odds ratio, CI; confidence interval, LV dysfunction; preoperative left ventricular dysfunction (ejection fraction < 40%), RBC; red blood cell.

**Supplementary Material 6. Risk factor analysis for failure-to-rescue (FTR).** The results of univariable and multivariable logistic regression analyses. Odds ratios with 95% confidence intervals and corresponding p values are presented

| **FTR** | **Univariable analysis** | | **Multivariable analysis** | |
| --- | --- | --- | --- | --- |
|  | **OR (95% CI)** | **P value** | **OR (95% CI)** | **P value** |
| Surgery type |  |  |  |  |
| Coronary |  |  |  |  |
| Valve | 2.04 (0.67-6.23) | 0.209 |  |  |
| Aorta | 1.41 (0.48-4.11) | 0.531 |  |  |
| **Male sex** | **0.35 (0.16-0.74)** | **0.007** | **0.30 (0.11-0.82)** | **0.018** |
| **Age** | **1.03 (0.99-1.06)** | **0.098** |  |  |
| **Resident absence** | **2.11 (0.99-4.49)** | **0.053** | **3.64 (1.33-9.98)** | **0.012** |
| Medical aid | 1.13 (0.24-5.37) | 0.875 |  |  |
| Body mass index |  |  |  |  |
| Normal  (≥18.5, <25) |  |  |  |  |
| Underweight (<18.5) | 1.76 (0.51-6.10) | 0.370 |  |  |
| **Overweight (≥25)** | **0.43 (0.18-1.03)** | **0.058** |  |  |
| Hypertension | 1.37 (0.56-3.36) | 0.486 |  |  |
| Diabetes mellitus | 1.73 (0.81-3.67) | 0.156 |  |  |
| Cerebrovascular accident | 1.30 (0.52-3.25) | 0.569 |  |  |
| **Chronic kidney disease** | **3.89 (1.76-8.59)** | **<0.001** | **4.33 (1.56-12.08)** | **0.005** |
| **Preoperative albumin** | **0.32 (0.17-0.58)** | **<0.001** |  |  |
| **LV dysfunction** | **7.32 (3.01-17.8)** | **<0.001** | **6.51 (2.00-21.16)** | **0.002** |
| Redo surgery | 0.92 (0.26-3.30) | 0.899 |  |  |
| **RBC transfusion** | **1.30 (1.19-1.43)** | **<0.001** | **1.31 (1.17-1.45)** | **<0.001** |
| **Complex surgery** | **1.97 (0.91-4.28)** | **0.086** |  |  |
| **EuroSCORE II** | **1.11 (1.05-1.16)** | **<0.001** |  |  |
| Elective surgery | 0.77 (0.35-1.70) | 0.521 |  |  |
| **Operation time** | **1.005 (1.002-1.008)** | **0.004** |  |  |

Abbreviations: FTR; failure-to-rescue, OR; odds ratio, CI; confidence interval, LV dysfunction; preoperative left ventricular dysfunction (ejection fraction < 40%), RBC; red blood cell.

**Supplementary Material 7. Number of staff and weekly working hours by occupation**

|  | **2023-Q1** | **2024-Q1** | **P value** | **2023-Q2** | **2024-Q2** | **P value** | **2023-Q3** | **2024-Q3** | **P value** |
| --- | --- | --- | --- | --- | --- | --- | --- | --- | --- |
| **Physician Assistant (PA)** | | | | | | | | | |
| **Number of staff (mean)** | 6 | 5.3 | - | 6 | 7 |  | 6 | 9 | - |
| **Weekly work hours** | 41 [40-42] | 42 [41-44] | 0.138 | 41 [40-44] | 43 [40-44] | 0.399 | 41 [40-42] | 40 [40-42] | 0.634 |
|  |  |  |  |  |  |  |  |  |  |
| **Surgical Assistant (SA)** | | | | | | | | | |
| **Number of staff (mean)** | 4 | 4 | - | 4 | 4 |  | 4 | 5.3 | - |
| **Weekly work hours** | 47 [45-50] | 51 [47-52] | 0.095 | 45 [43-48] | 52 [50-55] | **<0.001** | 47 [45-49] | 49 [43-51] | 0.437 |
|  |  |  |  |  |  |  |  |  |  |
| **Consultants** | | | | | | | | | |
| **Number of staff (mean)** | 7 | 7 | - | 7 | 6.6 |  | 7 | 6 | **-** |
| **Weekly work hours** | 60 [60-60] | 79 [60-87] | **<0.001** | 60 [60-60] | 81 [60-85] | **<0.001** | 60 [60-60] | 81 [80-82] | **<0.001** |

**Supplementary Material 8. End of the resident resignation**

As of September 1, 2025, approximately 65% of residents nationwide and 9 of 11 CT surgery residents in our institution had resumed training. The numerical discrepancy in our institution compared with earlier descriptions in the Methods section is due to two graduating chief residents completing training, one second-year resident entering mandatory military service, two of three newly appointed incoming residents declining their positions, and one second-year resident transferring from another hospital.

Among the 65% of the nation-wide residents who resumed their training, Dermatology, Ophthalmology, and Plastic Surgery specialties saw high rates, with 89.9% in Dermatology, 91.9% in Ophthalmology, and 89.4% in Plastic Surgery. Other specialties, such as radiology (91.5%), psychiatry (93.5%), and anaesthesiology (90.7%), also reported high rates, exceeding 90%. However, the high-risk specialties (internal medicine, general surgery, obstetrics and gynaecology, paediatrics, cardiothoracic surgery, and emergency medicine, etc.) that the government intended to foster had significantly lower rates, with internal medicine at 64.9%, surgery at 36.8%, obstetrics and gynaecology at 48.2%, and paediatrics at 13.4%. Emergency Medicine (42.1%) and Cardiovascular and Thoracic Surgery (21.9%).

Although the mass resignation was intended as political opposition to government policy rather than an effort to improve working conditions, it indirectly triggered significant systemic change. Public and political attention has increasingly focused on longstanding dependence on residents for service provision. This scrutiny led to indirect but meaningful improvements in trainee working conditions. The legally permitted weekly working hours for residents were reduced from 88 to 72 hours, and the maximum number of continuous duties was shortened from 36 to 24 hours. To make more efficient use of the reduced training hours, educational systems such as dry-lab and wet-lab training programs are also being discussed.
